# Diffraction order-engineered polarization-dependent silicon nano-antennas metagrating for compact subtissue Mueller microscopy

**DOI:** 10.1515/nanoph-2025-0405

**Published:** 2025-12-03

**Authors:** Qingyuan Li, Jianyao Li, Gaodi Chen, Zhiguang Lin, Dongmei Lu, Xiaoxu Deng

**Affiliations:** Department of Physics and Astronomy, 12474 Shanghai Jiao Tong University, Shanghai, 200240, China; Department of Hematology, Huashan Hospital, Fudan University, Shanghai, 200240, China; Department of Hematology, Division of Nutrition, Huashan Hospital, Fudan University, Shanghai, 200240, China

**Keywords:** metagrating, nanophotonics, polarization transformation, matrix Fourier optics, Mueller microscopy

## Abstract

A polarization-dependent silicon nano-antennas metagrating (PSNM) is proposed for parallel polarization transformation by engineering diffraction orders, upon which a compact Mueller microscopy system is implemented for subtissue-level polarization extraction. The polarization-dependent metagrating is designed using matrix Fourier optics and nonlinear optimization with four diffraction orders described by waveplate-like Jones matrices, which is encoded by nano-antennas combining geometric and propagation phases. The measured phase delay and orientation of each diffraction order of the metagrating deviate by less than 6.7 % from the design values, and the overall diffraction efficiency reaches 70.89 % with a coefficient of variation of 0.021. A transmissive PSNM Mueller microscopy system is developed by directly embedding the metagrating into an infinity-corrected microscopic optical path, which extracts subtissue-level polarization distributions of biological sections over a 152 μm × 152 μm field of view with reduced measurement redundancy, facilitating the differentiation and staging of pathological tissues for potential stain-free diagnostic applications.

## 1 Introduction

Polarization imaging reveals valuable microstructural details invisible to conventional microscopy, allowing observation of low-contrast, transparent biological tissues with anisotropic biomolecules [1], [2], [3], [4], [5]. Mueller matrix microscopy provides the most comprehensive microstructural information among polarization imaging techniques and is widely used in morphology and pathology [6], [7], [8], [9], especially for analyzing anisotropic changes in diseased tissues like cervical, skin, and other tumors [10], [11], [12], [13], [14], [15].

Optical metasurfaces offer precise modulation of light amplitude, phase, and polarization at subwavelength scales [16], [17], [18], [19], [20], [21], supporting applications such as versatile on-chip manipulation [22], [23], spin-controlled generation [24], full-color *in situ* imaging [25], chiral imaging [26], polarization holography [27], [28], [29], and Stokes imaging [30], [31]. Compact metasurface-based Stokes imaging has been proposed using interleaved or superpixel structures to spatially separate intensity information of different polarization states. Polarized subimages are spatially separated by interleaved metalenses, based on which full-Stokes imaging is reconstructed with an ultra-high numerical aperture (NA) [32]. By optimizing meta-atom lattice constant and scale of interleaved metalens, the imaging resolution, measured with a United States Air Force (USAF) test target, has reached 2.19 μm, with reduced electromagnetic crosstalk between interleaved sections. Orthogonal polarized components of incident image are selected and focused to corresponding pixel regions by superpixel structure, the intensities of which are detected superpixel by superpixel to reconstruct full Stokes imaging [33]. By trading spatial resolution, imaging polarimetry with a transmission efficiency of about 65 % is achieved using a superpixel configuration, overcoming the 50 % theoretical efficiency limit of traditional DOFP (division of focal plane) polarization cameras. Based on superpixel polarization imaging, metasurface-based Mueller microscopy is achieved by integrating ultra-thin multilayer nanograting filter arrays onto a CMOS sensor, providing exceptional compactness by eliminating redundant optical components and lengthy measurements typical of conventional systems, thereby facilitating advances in medical imaging and diagnostic science [34]. However, as the metasurface-based Mueller matrix microscopy is implemented using superpixels, each covering the area of six sensor pixels, the effective field of view is inherently restricted, resulting in reduced spatial resolution and increased vignetting [33], which in turn limits its applicability to subtissue-level imaging. Based on matrix Fourier optics [35], [36], [37], [38], a metagrating was designed using nanopillar arrays to control far-field polarization, achieving single-shot full-Stokes polarization camera for imaging distant objects without moving parts or specialized sensors [39], [40]. In this paper, a polarization-dependent silicon nano-antennas metagrating (PSNM) was proposed to achieve parallel polarization transformation without spatial multiplexing or a shared aperture, upon which a compact Mueller microscopy system was implemented for subtissue-level polarization imaging and anisotropy information extraction. Based on matrix Fourier optics, the metagrating is designed to preserve four diffraction orders with each possessing a Jones matrix in the form of a waveplate oriented in distinct directions. A nonlinear constrained optimization algorithm was employed to determine the phase profile of the designed 15 × 15 metagrating unit cell, achieving the desired Jones matrices in the retained diffraction orders with high and uniform diffraction efficiency. According to the optimized phase distribution, the metagrating structure was encoded by nano-antennas with both geometric and propagation phases. Then, the metagrating was fabricated on a fused silica substrate by EBL and ICP technique. Polarization and diffraction efficiencies of each fabricated metagrating order were experimentally measured, revealing maximum deviations of 6.7 % in phase delay and 4 % in fast axis orientation from the designed waveplates, with an overall diffraction efficiency of 70.89 % and a high uniformity quantified by a coefficient of variation of 0.021. By integrating the metagrating with a 100× objective lens, the transmissive PSNM Mueller microscopy system was constructed and eigenvalue-calibrated, featuring streamlined measurement procedures with a field of view (FOV) of 152 μm × 152 μm. Through Mueller matrix measurement conducted by the PSNM system and MMPD parameter extraction, the subtissue scale depolarization distribution of *Epipremnum aureum* leaf sections was measured, and the average depolarization was found to decrease with storage time, which is consistent with results obtained by conventional methods. Similar polarization analysis performed on mouse fibrotic liver and cervical cancer tissues identified total retardance as an effective metric for staging liver fibrosis and distinguishing cancerous from normal cervical tissue, highlighting the system’s potential application in portable, stain-free auxiliary cancer diagnosis and monitoring.

## 2 Design method

### 2.1 The mechanism for generating polarization response of diffraction orders

**Figure 1:**
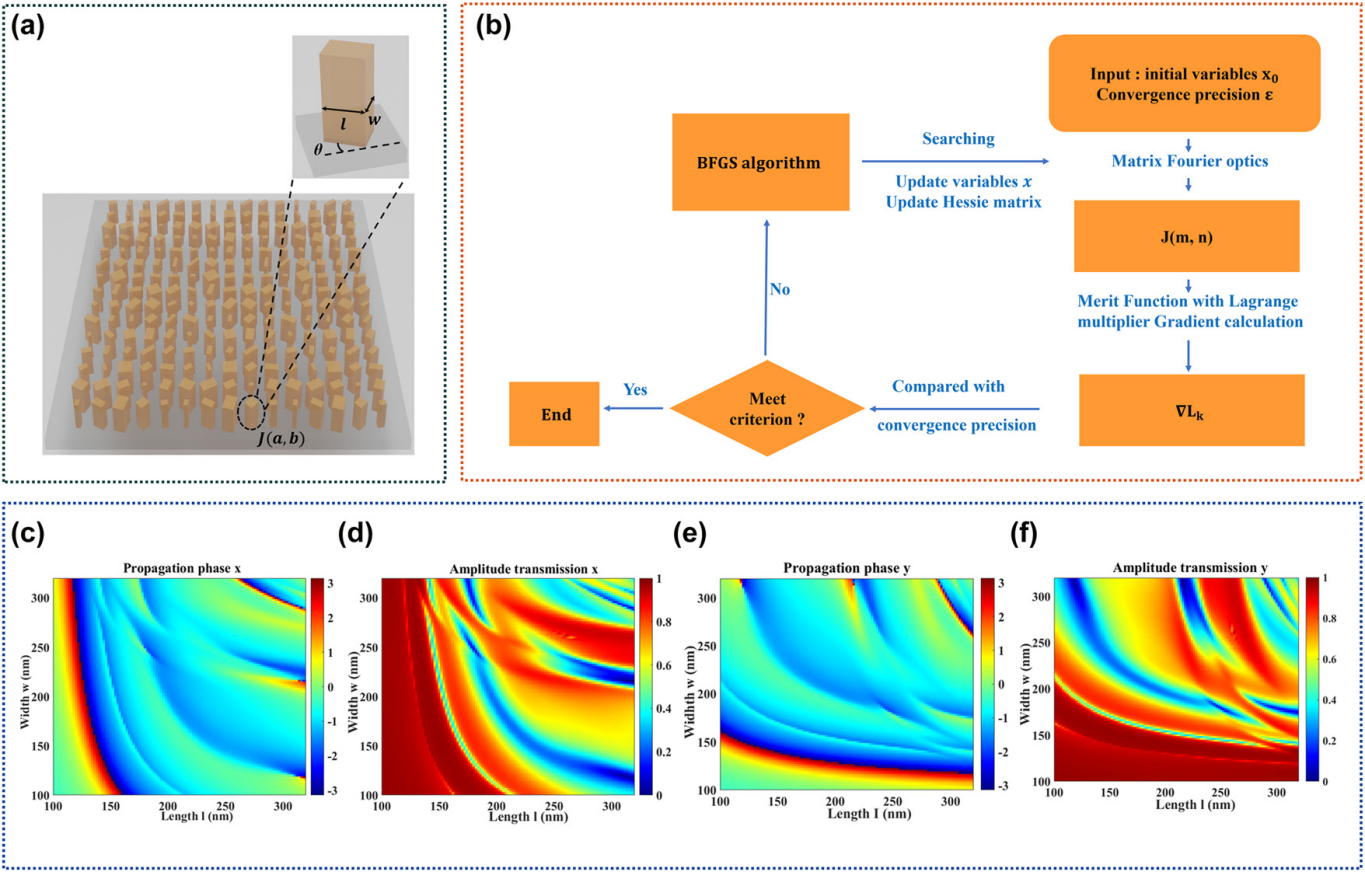
PSNM structure construction. (a) Unitcell schematic of PSNM and magnified structure diagram of nano-antennas. (b) The framework of structural optimization algorithm for nano-antennas polarization-dependent metagrating. (c), (d) Swept propagation phase and amplitude transmission with *x*-polarized incidence, and (e), (f): with *y*-polarized incidence.

The structure schematic of polarization-dependent silicon nano-antennas metagrating (PSNM) is shown in Figure 1(a). The nano-antenna has a rectangular cross section with length *l*, width *w*, and orientation angle *θ*. The unit cell of PSNM generally includes *p* × *q* nano-antennas with an interelement separation *d*. The Jones matrix of nano-antenna at row *a* and column *b* within a unit cell is given as:

(1) $$J\left(a,b\right)=R\left(\theta_{\left(a,b\right)}\right)\left[\begin{array}{cc} {\text{e}}^{\text{i}\varphi_{x}\left(a,b\right)} & 0\\ 0 & {\text{e}}^{\text{i}\varphi_{y}\left(a,b\right)} \end{array}\right]R\left(-\theta_{\left(a,b\right)}\right)$$

$R\left(\theta \right)$ is the 2 × 2 rotation matrix. Both *φ*_*x*_ and *φ*_*y*_ are the propagation phase adjusted by dimensions of the nano-antenna, and the geometric phase was controlled by orientation angle *θ*. Then the spatially dependent Jones matrix profile $J\left(x,y\right)$ of PSNM is derived:

(2) $$\begin{array}{ll} J\left(x,y\right) & =J_{\text{unitcell}}*\left[\frac{1}{pqd^{2}}comb\left(\frac{2x-pd}{2pd}\right)comb\right.\\ & \;\left.\times \left(\frac{2y-qd}{2qd}\right)\right] \end{array}$$

where:

$$\begin{array}{ll} J_{\text{unitcell}} & =\left[\overset{p}{\underset{a=1}{\sum }}\overset{q}{\underset{b=1}{\sum }}J\left(a,b\right)rect\left(\frac{2x-\left(2a-1\right)d}{2d}\right)rect\right.\\ & \;\left.\times \left(\frac{2y-\left(2b-1\right)d}{2d}\right)\right] \end{array}$$

$x\in \left[0,pd\right],\;rect\left(\frac{\left(2x-\left(2a-1\right)d\right)}{2d}\right)rect\left(\frac{\left(y-\left(2b-1\right)d\right)}{2d}\right)$ is the 2D-rectangle function centered at $\left(\frac{\left(2a-1\right)d}{2},\frac{\left(2b-1\right)d}{2}\right)$ . $comb\left(\frac{2x-pd}{2pd}\right)comb\left(\frac{2y-qd}{2qd}\right)$ is the 2D comb function; * is the convolution operator.

Illuminated by a normally incident uniform plane-wave $\left|E_{in}\right\rangle$ , the far-field of PSNM over angular coordinates $\left(k_{x},k_{y}\right)$ is given [39]:

(3) $$\left|A\left(k_{x},k_{y}\right)\right\rangle =\overset{+\infty }{\underset{-\infty }{\iint }}J\left(x,y\right)\left|E_{in}\right\rangle e^{-i\left(k_{x}x+k_{y}y\right)}dxdy$$

where $\left|E_{in}\right\rangle$ and $\left|A\left(k_{x},k_{y}\right)\right\rangle$ are the incident field and far-field in Jones vector representation. $\left|E_{in}\right\rangle$ is constant, which is taken out of the integral:

(4) $$J\left(k_{x},k_{y}\right)=\overset{+\infty }{\underset{-\infty }{\iint }}J\left(x,y\right)e^{-i\left(k_{x}x+k_{y}y\right)}dxdy$$

where $J\left(k_{x},k_{y}\right)\left|E_{in}\right\rangle =\left|A\left(k_{x},k_{y}\right)\right\rangle$ , $J\left(k_{x},k_{y}\right)$ is the Fourier transformation of $J\left(x,y\right)$ , representing the polarization-dependent response of different wave-vector component. Substituting Equations (2) into (4) and rearranging into a discrete form:

(5) $$\begin{array}{cc} J_{\left(m,n\right)} & =\frac{4\pi^{2}}{pq}\overset{p}{\underset{a=1}{\sum }}\overset{q}{\underset{b=1}{\sum }}J\left(a,b\right)sinc\left(\frac{m\pi }{p}\right)\\ & \;\times sinc\left(\frac{n\pi }{q}\right){\text{e}}^{\text{i}\pi \left(\frac{\left(2a-1\right)m}{p}+\frac{\left(2b-1\right)n}{q}\right)} \end{array}$$

*n* and *m* are integers. $J_{\left(m,n\right)}$ is the polarization-dependent response at diffraction order $\left(m,n\right)$ of PSNM. The metagrating achieves parallel polarization transformation across multiple diffraction orders without shared aperture or spatial multiplexing. From equation (5), the polarization response on each diffraction order of PSNM is fully determined by the phase profile and orientation distribution of nano-antennas.

### 2.2 Optimization and design implementation

The phase profile and orientation distribution of nano-antennas metagrating is designed by a nonlinear constrained optimization with respect to a figure of merit. The polarization-dependent metagrating is expected to retain the four diffraction orders $\left\{G\right\}=\left\{\left(\pm 1,0\right),\left(0,\pm 1\right)\right\}$ . The targeted polarization responses of the four diffraction orders are quarter wave-plates at different orientations angles $\theta_{\left(m,n\right),tar}$ with diffraction efficiencies as high as possible. The targeted Jones matrix of each retained order is:

(6) $$\begin{array}{ll} {\tilde{J}}_{\left(m,n\right),tar} & =R\left(\theta_{\left(m,n\right),tar}\right)\left[\begin{array}{cc} 1 & 0\\ 0 & i \end{array}\right]R\left(-\theta_{\left(m,n\right),tar}\right),\\ & \;\;\left(m,n\right)\in G \end{array}$$

The target orientation angles $\theta_{\left(0,\pm 1\right),tar}$ and $\theta_{\left(\pm 1,0\right),tar}$ of the Jones matrix corresponding to the retained diffraction orders of the metagrating are selected to exhibit mirror symmetry, i.e., $\theta_{\left(0,1\right),tar}=-\theta_{\left(0,-1\right),tar}$ and $\theta_{\left(1,0\right),tar}=-\theta_{\left(1,0\right),tar}$ , with values in the ranges of (±70°, ±80°) and (±40°, ±50°), respectively, which yield relatively low systematic error with a low condition number [41], [42]. The constraint conditions are: (1) the efficiencies are uniformly distributed across all retained diffraction orders. (2) The Jones matrix response on each retained order converges to the targeted form. Therefore, the optimization problem is generalized:

(7) where *F* is the merit function; superscript † represents the Hermitian conjugate, the trace $\mathrm{Tr}\left(J_{\left(m,n\right)}^{†}J_{\left(m,n\right)}\right)$ is the square of amplitudes of the complex entries in $J_{\left(m,n\right)}$ [35], represents the diffraction efficiency of the order $\left(m,n\right)$ . *σ* operator computes the standard deviation in the computed traces $\mathrm{Tr}\left(J_{\left(m,n\right)}^{†}J_{\left(m,n\right)}\right)$ , for all $\left(m,n\right)$ ϵ {*G*}. 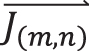 $={\left(z_{1},z_{2},z_{3},z_{4},z_{5},z_{6},z_{7},z_{8}\right)}^{T}$ is the 8 × 1 vector converted from 2 × 2 complex Jones matrix $J_{\left(m,n\right)}=\left[\begin{array}{cc} z_{1}+iz_{2} & z_{3}+iz_{4}\\ z_{5}+iz_{6} & z_{7}+iz_{8} \end{array}\right]$ , which is similar for 
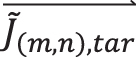
. *g*1 = 0 means the standard deviation of the diffraction efficiency among the retained diffraction orders is expected to be 0, to ensure that the “weights” of the Jones matrices $J_{\left(m,n\right)}$ in the desired orders are uniform. $g2_{\left(m,n\right)}=0$ means that the Jones matrices $J_{\left(m,n\right)}$ should converge to the targeted form ${\tilde{J}}_{\left(m,n\right),tar}$ during the optimization. Derived from equations (1)–(5), *F*, *g*1, *g*2_(*m*,*n*)_ are fully determined by metagrating variables $\varphi_{x}\left(a,b\right)$ , $\varphi_{y}\left(a,b\right)$ , and $\theta \left(a,b\right)$ .

Based on Lagrange multipliers method, the optimization problem equation (7) is transformed into an unconstrained optimization problem:

(8) $$\min\;L=1-F+\lambda^{T}g$$

where *λ* is the Lagrange multiplier vector, $g={\left[g1,g2_{\left(m,n\right)}\right]}^{T}$ , and *L* is the Lagrange function. The damped quasi-Newton algorithm with the Broyden–Fletcher–Goldfarb–Shanno (BFGS) update is employed to solve the isovalent unconstrained optimization problem as defined in equation (8) [43].

The framework of nano-antennas polarization-dependent metagrating optimization is shown as Figure 1(b):

Step 1: Set the polarization-dependent metagrating variables $\varphi_{x}\left(a,b\right)$ , $\varphi_{y}\left(a,b\right)$ , and $\theta \left(a,b\right)$ . Set algorithm parameters *λ* and convergence precision *ɛ*. Define the total optimization variable $x=\left[\varphi_{x}\left(a,b\right);\varphi_{y}\left(a,b\right);\theta \left(a,b\right);\lambda \right]$ .

Step 2: Start with an initial value of optimization variable $x_{0}=\left[\varphi_{x}^{0}\left(a,b\right);\varphi_{y}^{0}\left(a,b\right);\theta^{0}\left(a,b\right);\lambda_{0}\right]$ , then derive Jones matrix $J_{\left(m,n\right)}$ of each retained order from equations (1)–(5), and calculate Lagrange function at the initial step *L*_0_ from equation (8). Set the initial Hesse matrix *B*_0_ = *I*, where *I* is the identity matrix.

Step 3: Perform the damped quasi-Newton algorithm to iterate the metagrating structure variables. In the *k*-th iteration, the gradient of the Lagrange function at current step is ∇*L*_*k*_, gradient increment is *y*_*k*−1_ = ∇*L*_*k*_ − ∇*L*_*k*−1_, and last vector step length is *s*_*k*−1_ = *x*_*k*_ − *x*_*k*−1_. Update the approximate Hesse matrix inverse ${B_{k}}^{-1}$ under Broyden–Fletcher–Goldfarb–Shanno method with Woodbury formula implied:

(9) $$\begin{array}{ll} B_{k}^{-1} & =\left(I-\frac{s_{k-1}y_{k-1}^{T}}{y_{k-1}^{T}s_{k-1}}\right){B_{k-1}}^{-1}\left(I-\frac{y_{k-1}s_{k-1}^{T}}{y_{k-1}^{T}s_{k-1}}\right)\\ & \;+\frac{s_{k-1}s_{k-1}^{T}}{y_{k-1}^{T}s_{k-1}} \end{array}$$

where ***I*** is the identity matrix. By the quasi-Newton searching equation:

(10) $$d_{k}=-{B_{k}}^{-1}\nabla L_{k}$$

The search direction ***d***_***k***_ is determined. Then Wolfe linear search principle is applied:

(11) $$\left\{\begin{array}{c} L\left(x_{k}+a_{k}d_{k}\right)\leq L_{k}+\sigma_{1}\nabla L_{k}^{T}d_{k}\\ \nabla L{\left(x_{k}+a_{k}d_{k}\right)}^{T}d_{k}\geq \sigma_{2}\nabla L_{k}^{T}d_{k} \end{array}\right.$$

where $0<\sigma_{1}<\frac{1}{2},\sigma_{1}<\sigma_{2}<1$ . The optimized step length *a*_*k*_ in the *k*-th iteration is specified by equation (11). Then update the optimization variable *x*_*k*+1_ = *x*_*k*_ + *a*_*k*_ *d*_*k*_ of the next iteration.

Step 4: Repeat Step 3, until ∇*L*_*k*_ reaches the convergence precision ∇*L*_*k*_ ≤ *ɛ*, then output the total optimization variable $x^{op}=\left[\varphi_{x}^{op}\left(a,b\right);\varphi_{y}^{op}\left(a,b\right);\theta^{op}\left(a,b\right);\lambda \right]$ . The optimized metagrating variables $\varphi_{x}^{op}\left(a,b\right)$ , $\varphi_{y}^{op}\left(a,b\right)$ , and $\theta^{op}\left(a,b\right)$ are obtained.

During the optimization process, only $\theta_{\left(\pm 1,0\right),tar}=\pm 50^{◦}$ and $\theta_{\left(0,\pm 1\right),tar}=\pm 75^{◦}$ led to the fastest convergence of the damped quasi-Newton algorithm, as well as the most stable optimized metagrating phase parameters $\varphi_{x}^{op}\left(a,b\right)$ , $\varphi_{y}^{op}\left(a,b\right)$ , and $\theta^{op}\left(a,b\right)$ . Consequently, $\theta_{\left(\pm 1,0\right),tar}=\pm 50^{◦}$ and $\theta_{\left(0,\pm 1\right),tar}=\pm 75^{◦}$ are identified as the optimal target orientation parameters for equation (6).

The nano-antennas structure database was established by the numerical simulations, in which the amplitude transmission and propagation phase related to selected nano-antennas were also recorded. The height and lattice constant of α-Si nano-antennas were 710 nm and 550 nm, respectively. The length l and width w of each nano-antenna were swept in the range of 100 nm–320 nm with an interval of 2 nm at wavelength 808 nm. The propagation phase: $\varphi_{x}^{\text{database}}$ , $\varphi_{y}^{\text{database}}$ , and amplitude transmission: *t*_*x*_, *t*_*y*_ of sweeped nano-antenna were simulated by FDTD solution with both *x*-polarized and *y*-polarized plane wave incidence as shown in Figure 1(c)–(f). Only structures with amplitude transmission exceeding 0.9 were selected into the database, the phase of which were covered 0–2π.

The nano-antennas structures of the polarization-dependent metagrating unit cell were encoded according to the optimized phase profile. The mean square error $MSE_{\left(a,b\right)}$ between the propagation phase $\varphi_{x}^{\text{database}}$ , $\varphi_{y}^{\text{database}}$ of nano antennas in the database and optimized phase $\varphi_{x}^{op}\left(a,b\right)$ , $\varphi_{y}^{op}\left(a,b\right)$ is:

(12) $$\begin{array}{cc} MSE_{\left(a,b\right)} & ={\left|\exp\left(i\varphi_{x}^{op}\left(a,b\right)\right)-t_{x}\exp\left(i\varphi_{x}^{\text{database}}\right)\right|}^{2}\\ & \;+{\left|\exp\left(i\varphi_{y}^{op}\left(a,b\right)\right)-t_{y}\exp\left(i\varphi_{y}^{\text{database}}\right)\right|}^{2} \end{array}$$

At each spatial coordinate $\left(a,b\right)$ , the antenna with the minimal $MSE_{\left(a,b\right)}$ was selected. Then angular orientations of encoded α-Si nano-antennas was arranged in accordance with the optimized metagrating variable $\theta^{op}\left(a,b\right)$ . Thus, the structure of metagrating unit cell was obtained, and finally the metagrating was created by tessellating the unit cell.

## 3 Results

### 3.1 Metagrating characterization

#### 3.1.1 Simulation

**Figure 2:**
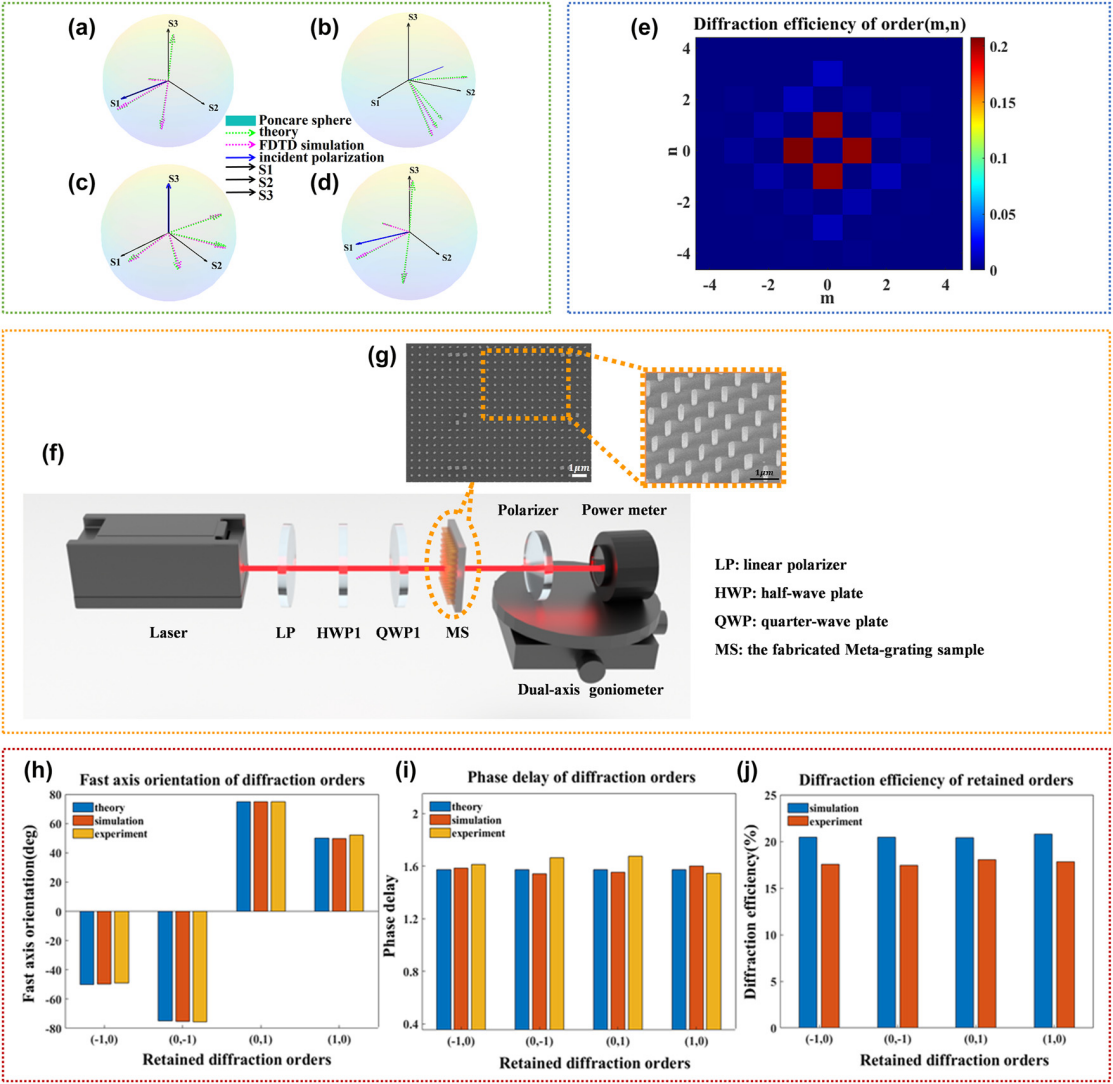
Simulation and characterization of PSNM: simulated/theoretical unified Stokes parameters of each retained diffraction orders under: (a) 0° linear polarized normal incidence, (b): elliptical polarized normal incidence, (c): right-circular polarized normal incidence, (d): 0° linear polarized; *θ* = 4°, *φ* = 45° oblique incidence. (e) The simulated diffraction efficiency of each order (*m*,*n*). (f) The experimental setup for the measurement of fabricated metagrating characterization. (g) The top view and magnified details of SEM images of the fabricated polarization-dependent silicon nano-antennas metagrating samples. (h) Theoretical, simulated, and experimental value of phase delay and fast axis orientation of each retained order. (i) Simulated and experimental diffraction efficiency of each retained diffraction order.

The polarization response of the four retained diffraction orders of the metagrating was simulated. The unit cell structure of metagrating was modeled in the Lumerical FDTD software with Periodical Boundary conditions under both normal incidence and oblique incidence at wavelength 808 nm. The polarization ellipse parameters of each retained diffraction order were monitored by a far-field polarization analysis group under linearly, circularly, and elliptically polarized incidence. The simulated polarization ellipse parameters, including azimuth angle *α*, ellipticity angle *ɛ*, and handedness *δ*, were converted to the unified Stokes parameters *S*_1_, *S*_2_, *S*_3_:

(13) $$\left\{\begin{array}{cc} S_{1} & =S_{0}\cos\left(2\alpha \right)\cos\left(2\varepsilon \right)\\ S_{2} & =S_{0}\sin\left(2\alpha \right)\cos\left(2\varepsilon \right)\\ S_{3} & =S_{0}\delta \sin\left(2\varepsilon \right) \end{array}\right.$$

where *S*_0_ refers to the first element of the Stokes vector. The unified Stokes parameters *S*_1_, *S*_2_, *S*_3_ of each retained diffraction order were simulated and displayed on the Poincaré sphere after being normalized by *S*_0_ as shown in Figure 2(a)–(d), which are consistent with the theoretical waveplate response under both normal and oblique incidence. The diffraction efficiency of each order, defined as the mean relative diffraction efficiency under orthogonal incident polarizations, was also simulated as shown in Figure 2(e). The diffraction efficiencies of the four retained diffraction orders were relatively uniform with an average efficiency of 20.53 % and a coefficient of variation of 0.0121. The simulated operational bandwidth of the nano-antennas metagrating is 780–835 nm, and the details of which are shown in the Supplementary Section S3.

The Mueller matrix is employed to further characterize the polarization responses of each diffraction order of the metagrating. The Mueller matrix is a 4 × 4 matrix that describes the linear transformation between the incident and transmitted Stokes vectors, characterizing the intrinsic polarization properties of the sample. The Mueller matrix of each retained diffraction order was simulated, from which the phase delay and fast axis orientation of each order was calculated. Under the *k*th independent incident polarization states $S_{k}^{in}$ , the polarization states $S_{k}^{\text{out}\left(\mathrm{m,n}\right)}$ of each retained diffraction order $\left(m,n\right)$ were simulated respectively. Then simulated Mueller matrix *M*_(*m*,*n*)_ of the metagrating diffraction order $\left(m,n\right)\in G$ was calculated:

(14) $$M\left(m,n\right)=O_{\left(m,n\right)}C^{T}{\left(CC^{T}\right)}^{-1}$$

where $C={\left[S_{k}^{in}\right]}_{k=1}^{16}$ ; $O_{\left(m,n\right)}={\left[S_{k}^{out\left(m,n\right)}\right]}_{k=1}^{16}$ . Both $S_{k}^{in}$ and $S_{k}^{out\left(m,n\right)}$ are 4 × 1 Stokes vectors, *C* and $O_{\left(m,n\right)}$ are both 4 × 16 matrix.

By employing the Mueller Matrix Polar Decomposition (MMPD) method [44], polarization parameters such as depolarization, total retardance, linear phase retardance, and linear fast axis orientation are extracted from the Mueller matrix. The waveplate’s birefringent behavior of each retained diffraction order is characterized by two parameters: the phase delay, which corresponds to the linear phase retardance, and the fast axis orientation, which corresponds to the linear fast axis orientation. Both parameters were shown as the red bars in Figure 2(g). Among four retained diffraction orders, the maximum deviation of phase delay between the simulated values and designed wave plate parameters was 2 %, and that of fast axis orientation was 0.81 %.

#### 3.1.2 Fabrication

The fused silica substrate was ultrasonically cleaned using acetone, isopropanol, and deionized water. A 710 nm thick α-Si film was deposited on the fused silica substrate by plasma-enhanced chemical vapor deposition (PECVD) with a 5 % mixture of silane in argon at 300 °C. A photoresist layer (ZEP 520A) with 170 nm thickness was spin-coated on the surface of α-Si film. The metagrating pattern was written in the photoresist layer via electro-beam lithography (EBL) at a beam current of 2 nA followed by development in a resist developer (ZED-N50, Zeon Chemicals). A chromium layer with 25 nm thickness was deposited on the patterned photoresist layer. Then the photoresist layer was lifted off and the metagrating pattern was transferred to the chromium layer as a hard mask. Finally, the single-layer α-Si metagrating sample was fabricated by inductively coupled plasma (ICP) etching technique and then cleaned with acetone, isopropanol, and deionized water.

#### 3.1.3 Fabricated metagrating characterization

The Mueller matrix of each retained diffraction order of the fabricated metagrating was experimentally measured. The experimental setup for the measurement of fabricated metagrating characterization is shown in Figure 2(f). Independent incident polarization states $S_{k}^{in}$ were generated by combinations of Half-Wave Plates (HWP) and Quarter-Wave Plates (QWP) at different angle orientations. The transmitted light of each retained diffraction order of the fabricated metagrating was analyzed under horizontal polarization, vertical polarization, 45° linear polarization, and left-handed circular polarization. Then the analyzed light intensity was measured by a photodiode sensor, from which the corresponding Stokes vector $S_{k}^{out\left(m,n\right)}$ was calculated. The experimental Mueller matrices of retained diffraction orders were obtained from equation (14), and experimental waveplate birefringent parameters of each diffraction order were deduced based on MMPD method, shown as the yellow bars in Figure 2(h) and (i). The maximum deviation of phase delay between the fabricated and designed metagrating was 6.7 % and that of fast axis orientation was 4 %, which is caused by fabrication errors of the metagrating. The diffraction efficiency of each order was determined by the mean value of the relative diffraction efficiencies under 0° and 90° linear polarization incidence, which was measured by an integrated power meter (PM200 power meter console and S140C power sensor, Thorlab) as shown in Figure 2(j). The experimental diffraction efficiencies of the retained orders were relatively uniform with coefficient of variation of 0.021, and an overall diffraction efficiency of 70.89 % was achieved. The measured absolute transmission efficiency of the fabricated metagrating was 62.8 %, which is lower than the simulated value of 86.2 %, primarily due to fabrication imperfections.

### 3.2 PSNM Mueller microscopy

#### 3.2.1 PSNM Mueller microscopy setup and calibration measurement

**Figure 3:**
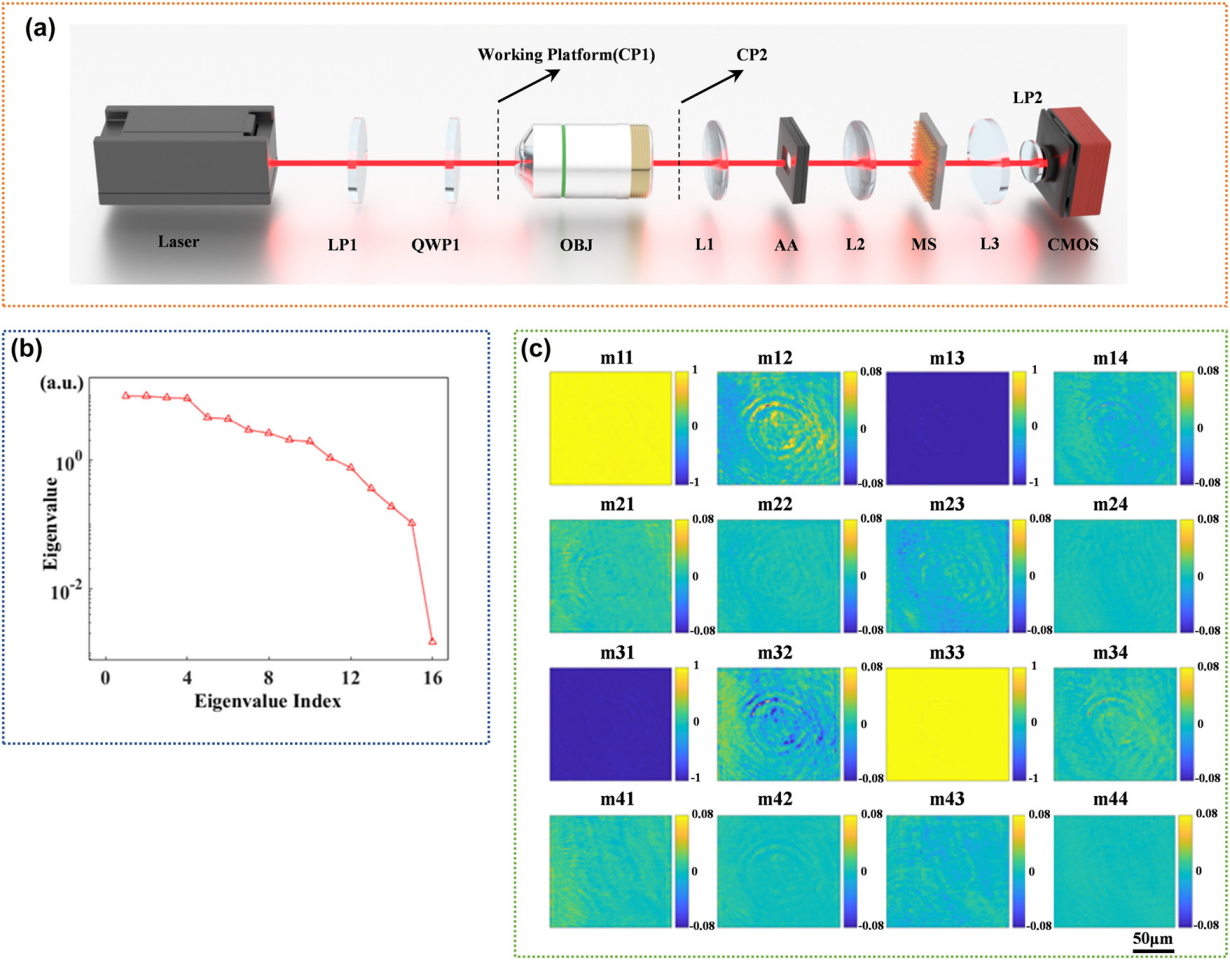
Calibration and verification of PSNM Mueller microscopy system. (a) The optical setup of the PSNM Mueller microscopy system. LP1 & LP2, linear polarizer, LP2 was SM1-mounted at the CMOS camera entrance; QWP1, quarter-wave plate; OBJ, microscope objective lens; L1 & L2 & L3, convex lenses (*f*1 = *f*2 = 100 mm, *f*3 = 25 mm); AA, adjustable aperture; MS, the fabricated metagrating sample; CP1 and CP2, calibration plane; CMOS, complementary metal oxide semiconductor. (b) The experimental eigenvalues *λ*_1_, *λ*_2_ … … to *λ*_16_ of *K*. (c) Normalized Mueller matrix image of the verification sample.

**Figure 4:**
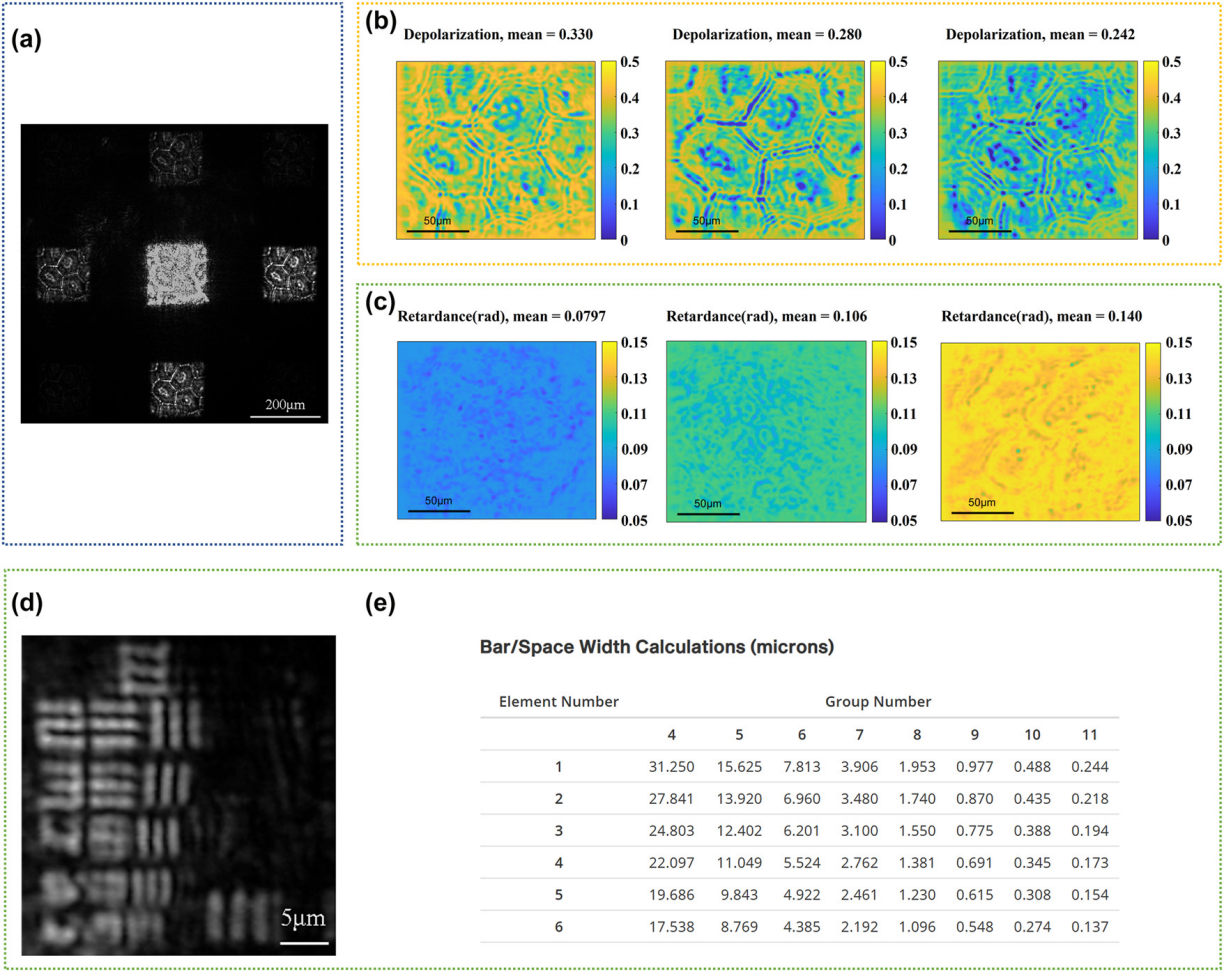
Resolution measurement and biological tissue characterization of the calibrated PSNM Mueller microscopy system. (a) Original intensity image captured by the COMS camera while the orientation angle of the quarter waveplate of the PSNM Mueller microscopy system was 75°, the bright central region originated from stray light, and the defocused image generated by zero-order light, which did not interact with the metagrating. (b) Depolarization distribution obtained by MMPD and the corresponding mean value of the fresh-cut tissue sections of *Epipremnum aureum* leaf measured at different time points. From left to right: 0 h, 3 h, and 6 h. (c) Total retardance distribution as well as the corresponding mean value for each stage of fibrotic tissue sections. From left to right: F0 (normal), F2 (moderate fibrosis), and F4 (Cirrhosis). (d) The image of the eighth group pattern of the resolution test target (HIGHRES-1, Newport). (e) Correspondence table of feature line width for elements in each group of the USAF resolution test target (HIGHRES-1, Newport).

The optical setup of the PSNM Mueller microscopy system was shown in the Figure 3(a). The metagrating was placed between the objective lens (HC FL PLAN 100×, Leica, FOV = 250 μm in diameter) and CMOS camera. The laser wavelength used in our experiments is 808 nm, which offers superior transmittance for biological tissues. The resolution of the PSNM Mueller microscopy system was 1.55 μm, which was measured through a USAF test target (HIGHRES-1, Newport). As shown in Figure 4(d), the image of the eighth group pattern of the resolution test target was measured by the Mueller microscopy system. The elements in the third row are clearly resolved, whereas those in the fourth row are only partially distinguishable. Based on the table in Figure 4(e), the line width of the third-row element in Group 8 is 1.55 μm; therefore, the spatial resolution of our system has reached at least 1.55 μm. The FOV of each diffraction channel was 152 μm × 152 μm effectively preserving the maximal inscribed square region within the objective lens’s original FOV.

The system was then calibrated and verified. The calibration samples were a quarter waveplate of which the preset fast axis orientation was about 30°, a linear polarizer (#OCZ200, BOCIC; theoretical transmission 78 %) of which the azimuth angle were preset to approximately 0° and 45°, respectively. Verification sample for PSNM Mueller microscopy system was a linear polarizer with preset azimuth angle about −45°. The samples, including the calibration samples and the verification sample, were individually positioned on the working platform of the microscope objective lens. The orientation angle of the quarter waveplate QWP1 of the PSNM Mueller microscopy system was changed (in our experiment were 50°, 75°, 105°, and 130°, respectively) to control the polarization incidence. The intensity distributions of four retained diffraction channels of the PSNM Mueller microscopy system were captured by the CMOS camera under different polarization incidence as $I_{c\text{\_}i_{\gamma }}^{\beta }$ for calibration samples and $I_{v_{\gamma }}^{\beta }$ for the verification sample (*β* represents different polarization incidence and *γ* represents different retained diffraction orders). Finally the 4 × 4 intensity projection matrix of the calibration samples and the verification sample were recorded as *D*_*c*_*i*_ and *D*_*v*_, of which matrix elements were expressed as: $D_{c\text{\_}i}\left(\beta ,\gamma \right)=I_{c\text{\_}i_{\gamma }}^{\beta };D_{v}\left(\beta ,\gamma \right)=I_{v_{\gamma }}^{\beta }\left(\beta ,\gamma =1,2,3,4\right)$ . The null response of the system *D*_0_, which referred to the intensity projection matrix without samples, was also measured in the same method.

#### 3.2.2 Data analysis

The Eigenvalue Calibration Method (ECM) [45] was adapted to calibrate the PSNM Mueller microscopy system. Based on the measured intensity projection matrices, the Sylvester equations of each calibration sample were summed:

(15) $$\sum {H_{i}}^{T}H_{i}W\text{\_}\text{vec}=KW\text{\_}vec=0$$

where $H_{i}=M_{c\text{\_}i}⊗E-E⊗{\left({D_{0}}^{+}D_{c\text{\_}i}\right)}^{T}$ represents the linear mapping of the vectorized Sylvester equation, ⊗ is the Kronecker product operator, superscript + represents the Moore–Penrose pseudo-inverse, *E* is the 4 × 4 identity matrix, *K* is a positive semidefinite Hermitian matrix, *M*_*c*_*i*_ is Mueller matrix of each calibration sample, *W*__vec_ is the reshaped 16 × 1 vector form of the 4 × 4 modulation matrix *W*, which represents the projection matrix of the generated input Stokes vectors. *K* is a positive semidefinite Hermitian matrix, *M*_*c*_*i*_ is Mueller matrix of each calibration sample. The eigenvalues of *K* are denoted as *λ*_1_, *λ*_2_ … … to *λ*_16_ from large to small, and *W*_vec is the eigenvector corresponding to null eigenvalue *λ*_16_. Based on the measured intensity projection matrices of the calibration samples and null response, the characteristics of calibration sample were determined through optimization by equation (15). Minimizing $\frac{\lambda_{16}}{\lambda_{15}}$ was taken as the optimization objective, driving *λ*_16_ toward the theoretical null value. The preset values of the characteristics of calibration samples were employed as initial values for the optimization process. The steepest descent algorithm was applied to optimize the characteristics of calibration samples: the orientation of the quarter wave plate was 29.61°, the orientation angles of the polarizer were 0.19° and 44.35°, and the transmittance of the polarizer was 77.34 %. Then *K* was also determined from equation (15), and the eigenvalues (shown in the Figure 3(b)) and the corresponding eigenvectors were calculated from the diagonalization of *K*. The value of *λ*_16_, which was theoretically null eigenvalue, reached the order of 10^−3^. Modulation matrix *W* of the PSNM Mueller microscopy system was obtained by rearranging *W*_vec into 4 × 4 form, and the instrument matrix *A* of the system was deduced from: *A* = *D*_0_∗*W* ^−1^. The detailed deviation is shown in Supplementary Section S2.

The Mueller matrix distribution $M_{v}\left(x,y\right)$ of the verification sample was derived from the measured intensity projection matrix distribution $D_{v}\left(x,y\right)$ by:

(16) $$D_{v}\left(x,y\right)=AM_{v}\left(x,y\right)W$$

which is shown in Figure 3(c). The maximal deviation of the mean Mueller matrix element of the verification sample compared with the theoretical one was as small as 0.0094, primarily attributed to the random noise in the captured light intensities. Therefore, the calibration of the PSNM Mueller microscopy system was completed and validated.

The air sample was also measured for verification based on a backward eigenvalue calibration. In the backward calibration, the sample is placed on the calibration plane CP2. The instrument matrix *A* is derived through a procedure similar to that used for the modulation matrix *W*, rather than directly from the null response. The Mueller matrix of the air sample was determined using equation (16) by employing the modulation matrix *W*, the backward calibration-determined *A*, and the null response *D*_0_. The maximal deviation of the mean Mueller matrix element of the air sample compared with the theoretical value was 0.0143, which is comparable to previously reported high performance system [46], [47]. The details of air sample verification are shown in Supplementary Section S2.

#### 3.2.3 Polarization characteristics of biological tissue explored by calibrated PSNM Mueller microscopy system

The calibrated PSNM Mueller microscopy system was used to investigate the subtissue-level polarization distributions of various biological samples.

The fresh-cut tissue sections of *E. aureum* leaf were placed at the working plane position in the experimental setup shown as Figure 3(a). The intensity projection matrix distribution was measured using the calibrated PSNM Mueller microscopy system at three distinct time points: immediately after sample preparation (0 h), and subsequently at 3-h and 6-h intervals, following the same protocol as outlined in Section 3.2.1. Then the Mueller matrix distribution of *E. aureum* leaf section was calculated from equation (16) as shown in Supplementary Figure S5. Based on the MMPD method mentioned in Section 3.1.1, the measured Mueller matrix distributions were analyzed to extract polarization parameters at each time point, among which the depolarization parameter exhibited a pronounced change, as shown in Figure 4(b). The average depolarization decreased over storage time due to variations in the water content of plant leaves, a trend which is consistent with results obtained from conventional Mueller microscopy [48].

The polarization characteristics of fibrotic tissue sections from mouse liver were subsequently measured by the calibrated PSNM Mueller microscopy system. The Mueller matrix distribution for normal stage F0, moderate liver fibrosis stage F2, and cirrhosis stage F4 of the unstained tissue paraffin sections were measured as shown in the Supplementary Figure S6. Among the polarization parameters extracted by the MMPD method mentioned in Section 3.1.1, the total retardance parameter exhibited substantial changes across different stages of fibrosis, as shown in Figure 4(c). The progression of tissue fibrosis stages led to an increase in the mean of retardance distribution; therefore, the total retardance parameter is considered as a potential polarization-based metric for characterizing the stages of liver fibrosis.

**Figure 5:**
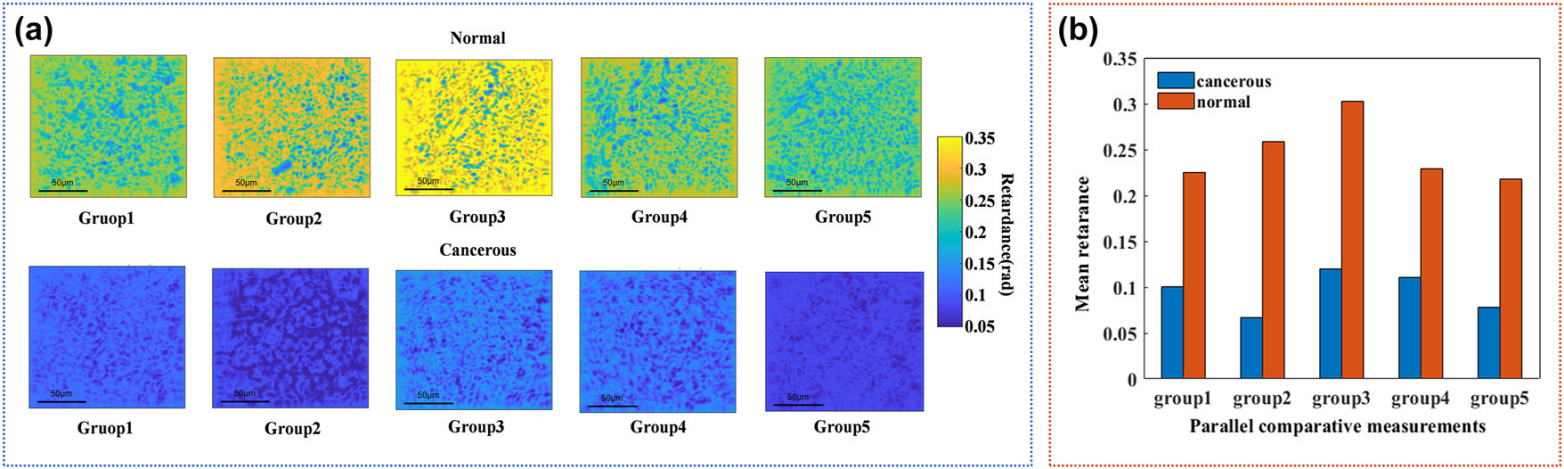
Comparative polarization analyses between cancerous and normal cervical tissue sections. (a) The total retardance distribution of each parallel experiment group. The first column represented cancerous cervical tissues from each group, while the second column represented normal tissues. (b) The mean of total retardance of normal and cancerous cervical tissues from each parallel experimental group.

Mueller matrix measurements and MMPD parameter extraction were also conducted using the calibrated PSNM Mueller microscopy system to characterize the polarization properties of mouse cervical cancer tissue. Comparative polarization analyses were performed on parallel experimental groups, each containing one cancerous tissue section and one normal tissue section. Among the polarization parameters extracted from the Mueller matrix distributions of each parallel experimental group, the total retardance was identified as a highly effective parameter for distinguishing between cancerous and normal tissue sections as shown in the Figure 5(a). Normal cervical tissue groups were characterized by a considerably higher mean total retardance than cancerous tissue groups as shown in the Figure 5(b), since tumor-associated fibrosis was a common phenomenon during cancer progression [49], [50], [51]. The calibrated PSNM Mueller microscopy system facilitated detailed polarization analysis from tissue sections, holding promise in cancer diagnosis.

## 4 Discussion

The single-layer metagrating achieved microscopic imaging with separated polarization control via engineered waveplate responses at designated diffraction orders without shared aperture or spatial multiplexing, upon which a compact Mueller microscopy system was facilitated. Despite fabrication imperfections in the metagrating, the PSNM Mueller microscopy system maintained high measurement precision after appropriate calibration and reduced the measurement requirements due to the parallel design. The experiment was conducted at the wavelength of 808 nm, within a spectral region recognized for its effective penetration in biological tissue. By choosing a waveplate as the target Jones matrix for each diffraction order, more optical energy can be transmitted into the imaging system, which is particularly advantageous for detecting weak transmitted images from biological tissues. Moreover, the Jones matrix of each diffraction order of the metagrating can also be engineered to emulate the responses of various polarization elements, which in turn enables the realization of integrated and parallel polarization imaging systems designed for specific functionalities, such as polarization remote sensing [52] and robot perception [53].

In conclusion, a polarization-dependent silicon nano-antennas metagrating (PSNM) was proposed for parallel polarization transformation, facilitating the development of a compact PSNM Mueller microscopy system with reduced measurement requirements. Based on matrix Fourier optics, the metagrating was engineered to yield quarter-waveplate-like Jones matrices with distinct orientations in the retained diffraction orders. Nonlinear constrained optimization was applied to ensure uniformity and high overall efficiency across these orders. Experimental characterization of the fabricated metagrating revealed maximum deviations of 6.7 % in phase delay and 4 % in fast axis orientation from the designed waveplates, with an overall diffraction efficiency of 70.89 % and a high uniformity quantified by a coefficient of variation of 0.021. The metagrating was employed to implement a compact Mueller matrix microscopy system with a 152 μm × 152 μm FOV and 1.55 μm resolution. The maximal deviation between the experimentally averaged and theoretical Mueller matrix elements of the verification sample was as low as 0.0094. The PSNM-based Mueller matrix measurements and MMPD parameter extraction conducted on *E. aureum* leaf sections have revealed a storage-time-dependent decrease in average depolarization consistent with conventional results. Similar polarization analysis performed on mouse fibrotic liver and cervical cancer tissues has identified total retardance as a promising metric for staging liver fibrosis and distinguishing cancerous from normal cervical tissue, highlighting the system’s potential for application in stain-free cancer diagnosis.

## Supplementary Material

Supplementary Material Details
