# Supplementary material for: Diffraction order-engineered polarization-dependent silicon nano-antennas metagrating for compact subtissue Mueller microscopy
Source: Nanophotonics. 2025 Dec 3;14(27):5277–89. doi: 10.1515/nanoph-2025-0405 (PMC12717920; doi:10.1515/nanoph-2025-0405)
Supplement: Supplementary file 1 — Supplementary Material Details [file j_nanoph-2025-0405_suppl_001.pdf]

Supporting information

# Diffraction order–engineered Silicon Nano-antennas Polarization-dependent Metagrating for Compact Subtissue Mueller Microscopy

*Qingyuan Li,*[†] *Jianyao Li,*[†] *Gaodi Chen,*[†] *Zhiguang Lin,*[‡] *Dongmei Lu,*[¶] *and Xiaoxu Deng*[*†]

†Department of Physics and Astronomy, Shanghai Jiao Tong University, Shanghai, 200240, China

‡Department of Hematology, Huashan Hospital, Fudan University, Shanghai, 200240, China

¶Department of Hematology, Division of Nutrtion, Huashan Hospital, Fudan University, Shanghai, 200240, China

## S1. Diffraction efficiency of the metagrating-based Mueller matrix microscopy system

The diffraction efficiency of the metagrating-based Mueller matrix microscopy system was measured under orthogonal polarization illumination in the presence and absence of the Epipremnum aureum leaf section. After removing the linear polarizer LP2 in the experimental setup as shown in Fig. 3(a), the optical power of each diffraction channel is measured under 0° and 90° linearly polarized incidence. The diffraction efficiency of each channel is shown in Fig. S1, determined in the same approach mentioned in section 3.1.3. The overall diffraction efficiency of the metagrating-based Mueller matrix microscopy system without sample has decreased to 66.4%, and the absolute transmission efficiency has been dropped to 46.1% owing to the integration loss. After placing the Epipremnum aureum leaf section at the working platform of the system, the overall diffraction efficiency of the metagrating-based

Mueller matrix microscopy system has decreased to 54.7%, and the zeroth order exhibited a further increase due to the depolarization effects of the sample.

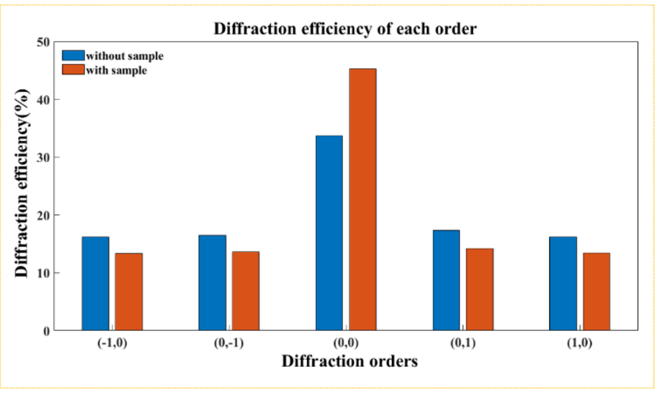


**Fig. S1:** Experimental diffraction efficiency of each diffraction order channel of the metagrating-based Mueller matrix microscopy system under sample-present and sample-absent conditions.

.

## S2 Eigenvalue calibration deviation and air sample verification results

The calibration of the metagrating-based Mueller matrix microscopy system is performed by placing the calibration sample on the working platform which is labeled as the calibration plane CP1 as shown in Fig.S2(a). The measured intensity projection matrix $D_{c\_i}$ is denoted as:

$$D_{c\_i} = AM_0{}'M_{c\_i}W \qquad (s.1)$$

where $M_0{}' = M_{obj}M_0$, $M_{obj}$ represents the Mueller matrix of microscope objective lens, $M_0$ represents the Mueller matrix of air, $M_{c\_i}$ is Mueller matrix of each calibration sample, $W$ is the modulation matrix of the system which represents the projection matrix of the generated input Stokes vectors, $A$ is the instrument matrix of the system [45]. To eliminate matrices $A$ and $M_0{}'$ in equation $(s.1)$, the comparison matrix $C_{c\_i}$ of each calibration sample is introduced:

$$C_{c\_i} = D_0{}^{+}D_{c\_i} = W^{+}M_0{}'^{+}A^{+}AM_0{}'M_{c\_i}W = W^{+}M_{c\_i}W \qquad (s.2)$$

where the matrix with the superscript $+$ denotes the Moore-Penrose pseudo-inverse matrix, $D_0$ is the null response of the system. Left-multiplying equation (s.2) by $W$ yields the Sylvester equations for each sample in terms of $W$:

$$M_{c\_i}W - WC_{c\_i} = 0 \qquad (s.3)$$

Equation $(s.3)$ is rewritten to the vectorized expression:

$$(M_{c\_i} \otimes E - E \otimes C_{c\_i}{}^T)W_{\_vec} = H_i W_{\_vec} = 0 \qquad (s.4)$$

where $W_{\_vec}$ is the $16 \times 1$ vector reshaped from the $4 \times 4$ modulation matrix $W$, $H_i = M_{c\_i} \otimes E - E \otimes \left(D_0{}^+ D_{c\_i}\right)^T$ represents the linear mapping of the vectorized Sylvester equation, $E$ is the $4 \times 4$ identity matrix. The Hermitian form of equation$(s.4)$ is:

$$H_i{}^T H_i W_{\_vec} = 0 \qquad (s.5)$$

where the superscript $T$ is the transpose operator. By summing the equation $(s.5)$ of all calibration samples, the equation (15) in the manuscript is obtained:

$$\sum H_i{}^T H_i W_{\_vec} = K W_{\_vec} = 0 \qquad (\mathrm{s.}6)$$

where $K$ is a positive semidefinite Hermitian matrix. $K$ is diagonalized as:

$$K = O^T \begin{bmatrix} \lambda_1 & \cdots & 0 \\ \vdots & \ddots & \vdots \\ 0 & \cdots & \lambda_{16} \end{bmatrix} O \qquad (s.7)$$

where the eigenvalues of $K$ are denoted as $\lambda_1$、$\lambda_2$…… to $\lambda_{16}$ from large to small; $O$ is the orthogonal matrix, the row vectors of which are the eigenvectors of $K$. $W_{\_vec}$ is derived from the row vector corresponding to $\lambda_{16}$ in $O$:

$$W_{\_vec} = O^T \begin{bmatrix} 0 \\ 0 \\ \vdots \\ 1 \end{bmatrix} \quad (s.8)$$

The backward calibration of the metagrating-based Mueller matrix microscopy system is performed by placing the calibration sample on the calibration plane CP2 as shown in Fig.S2(a). The measured backward intensity projection matrix $D_{b\_i}$ is denoted as:

$$D_{b\_i} = AM_{c\_i}M_0{}'W \quad (s.9)$$

Compared with equation $(s.1)$, the multiplication order of matrices $M_0{}'$ and $M_{c\_i}$ is exchanged in equation $(s.9)$ induced by the backward calibration. Therefore matrices $W$ and $M_0{}'$ are eliminated simultaneously by introducing the backward comparison matrix $C_{b\_i}$ of each calibration sample:

$$C_{b\_i} = D_{b\_i}D_0{}^+ = AM_{c\_i}M_0{}'WW^+M_0{}'^+A^+ = AM_{c\_i}A^+ \quad (s.10)$$

Right-multiplying equation $(s.10)$ by $A$ yields the Sylvester equations for each sample in terms of $A$:

$$AM_{c\_i} - C_{b\_i}A = 0 \quad (s.11)$$

Similarly, vectorization and summation are applied to construct Hermitian matrix $K'$:

$$\sum H_i'^T H_i' A_{\_vec} = K'A_{\_vec} = 0 \quad (s.12)$$

where $H_i' = C_{bi} \otimes E - E \otimes M_{c\_i}{}^T$. $A_{\_vec}$ is the 16×1 vector reshaped from the 4×4 instrument matrix, which is determined from the diagonalization of $K'$.

When air is used as the verification sample, the corresponding intensity projection matrix is the null response $D_0$ . According to equation (16), $D_0 = AM_0W$, the Mueller matrix of air $M_0$ is derived as shown in Fig.S2 (b). The maximal deviation of the mean Mueller matrix element of the air sample compared with the theoretical value was

0.0143. The maximal deviation of our system is comparable to previously reported high performance system, which is around 0.01[46], [47].

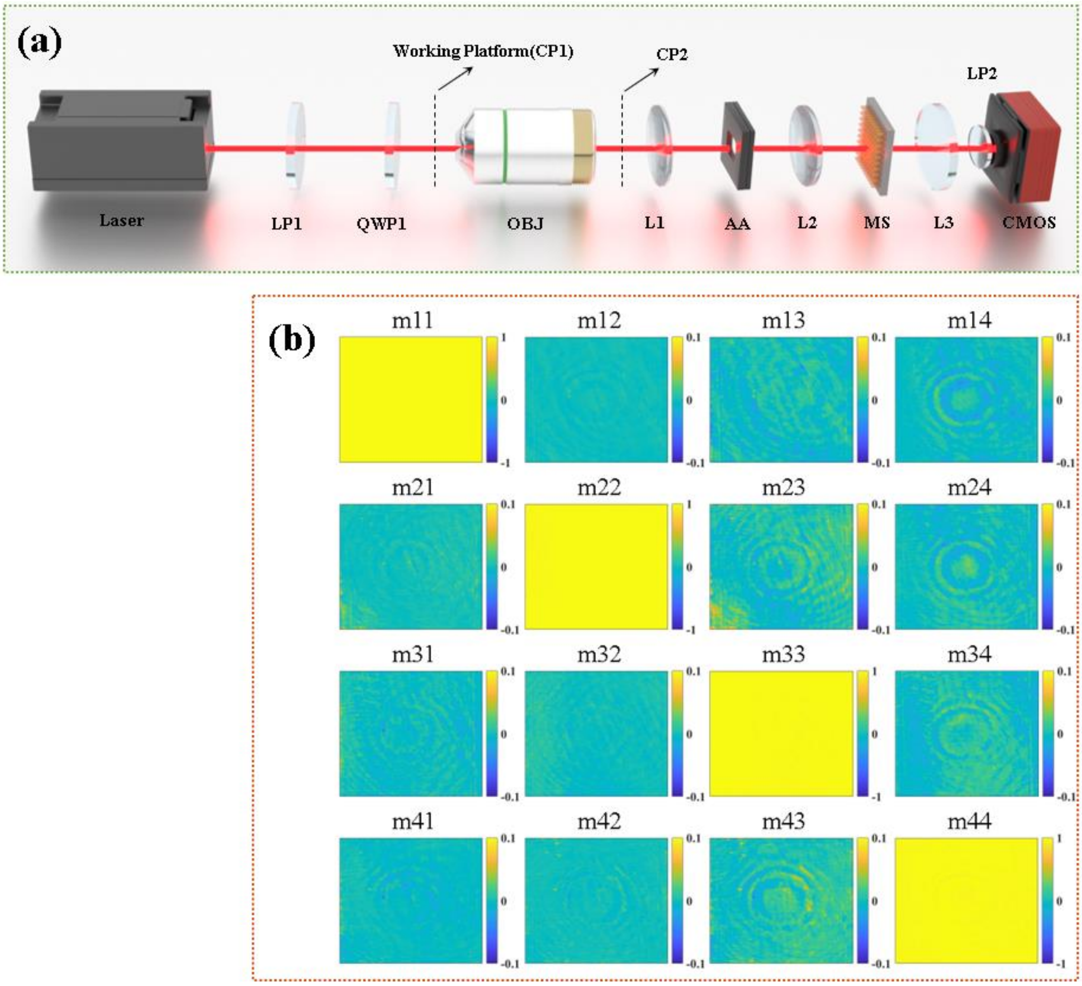


**Fig. S2:** (a) Schematic diagram of the backward calibration measurement for air sample verification. CP1 and CP2, the calibration planes. (b) Normalized Mueller matrix image for air sample verification.

**S3 Operational bandwidth simulation**

The operational bandwidth of the proposed polarization-dependent silicon nano-antennas metagrating (PSNM), defined as the wavelength range over which the overall diffraction efficiency falls to 80% of the value at 808 nm, is determined by FDTD simulations. The simulated overall diffraction efficiency of the nano-antennas metagrating drops to 66.86% at 780 nm and 66.58% at 835 nm, which are close to 80% of that at 808 nm (82.12%). Therefore, the simulated operational bandwidth is 780–835 nm. The corresponding simulation results are shown in Figures S3 and S4.

The zeroth-order diffraction is not observed in the simulation result of the metagrating at designed wavelength 808 nm, and the coefficient of variation in diffraction efficiencies of the retained diffraction orders is 0.0121 as shown in Fig. 2(e). The deviation of operating wavelength from the designed wavelength leads to the

occurrence of the zeroth order at 780 nm and 835 nm with diffraction efficiencies of 8.5% and 10.1%, respectively. Although the coefficients of variation in diffraction efficiencies of the retained diffraction orders increase to 0.0353 and 0.0162 at 780 nm and 835 nm, respectively, the diffraction efficiency of retained orders at both wavelengths remain relatively uniform, as shown in Fig S3(a) and Fig S4(a).

The comparison between the simulated and theoretical waveplate parameters for each diffraction order of the metagrating at 780 nm and 835 nm is shown in Fig.S3(b), (c) and Fig.S4(b), (c). The maximum deviation of the fast-axis orientation reaches 6% and the maximum deviation of the retardation is 4.9% at 780nm. At 835 nm, these deviations are 5% and 1.9%, respectively. The maximum deviations at 758 nm and 858 nm are larger than those at 808 nm, while still remaining relatively low.

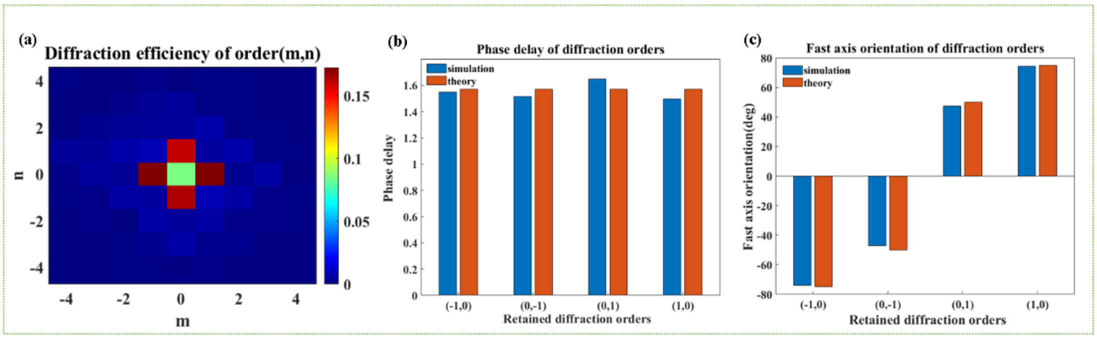


**Fig S3:** Simulation of PSNM at 780nm. (a) The simulated diffraction efficiency of each order (m,n). (b) Theoretical and simulated value of phase delay of each retained order. (c) Theoretical and simulated value of fast axis orientation of each retained order.

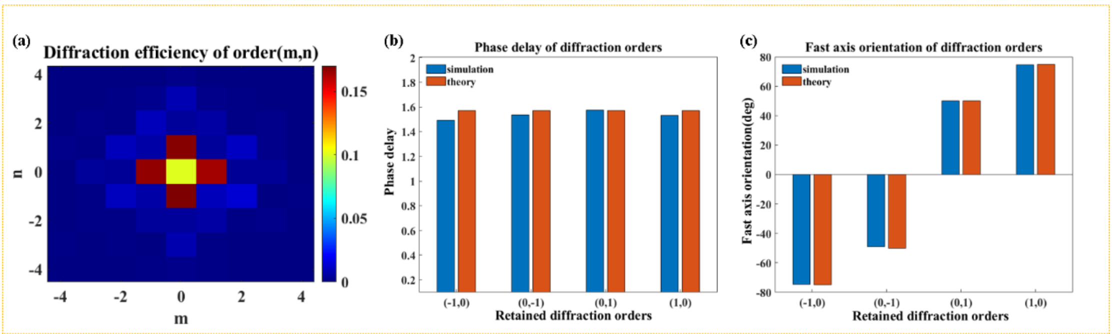


**Fig S4:** Simulation of PSNM at 835nm. (a) The simulated diffraction efficiency of each order (m,n). (b) Theoretical and simulated value of phase delay of each retained order. (c) Theoretical and simulated value of fast axis orientation of each retained order.

**0h:**

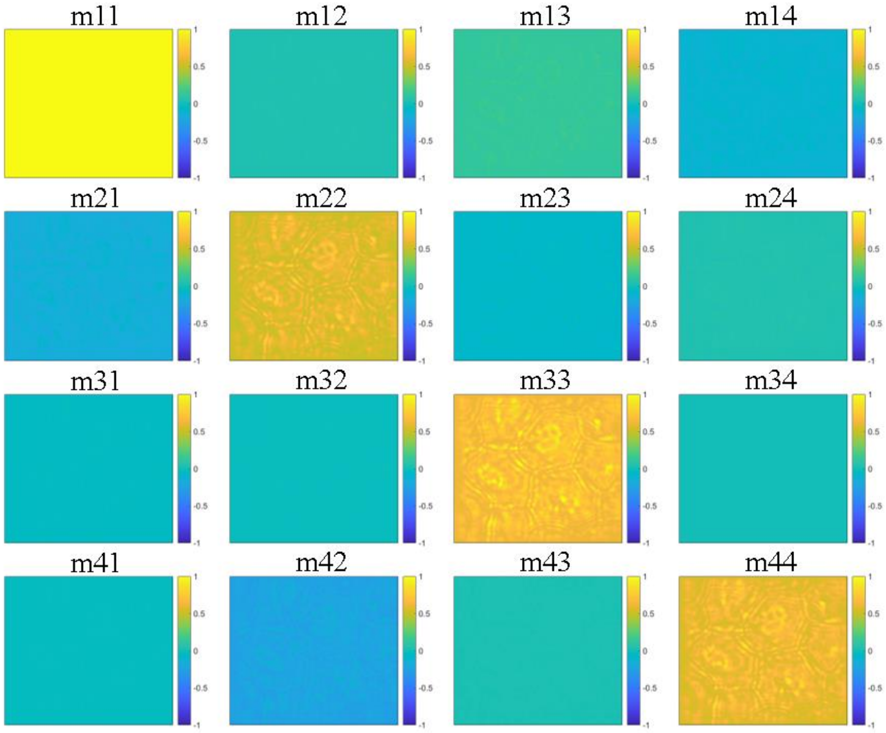
m11
m12
m13
m14
m21
m22
m23
m24
m31
m32
m33
m34
m41
m42
m43
m44

**3h:**

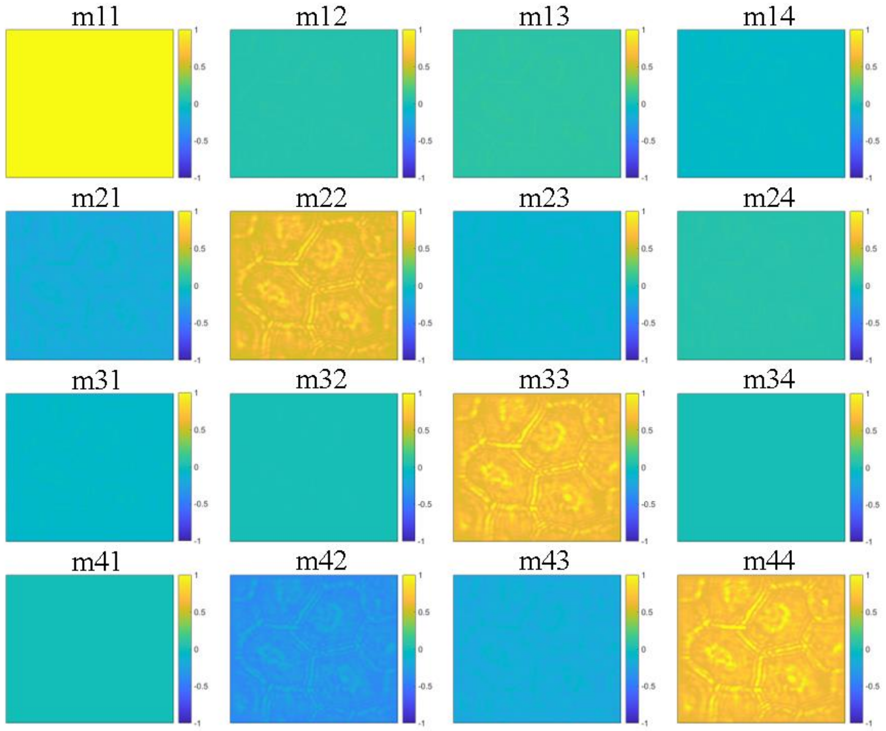
m11
m12
m13
m14
m21
m22
m23
m24
m31
m32
m33
m34
m41
m42
m43
m44

**6h:**

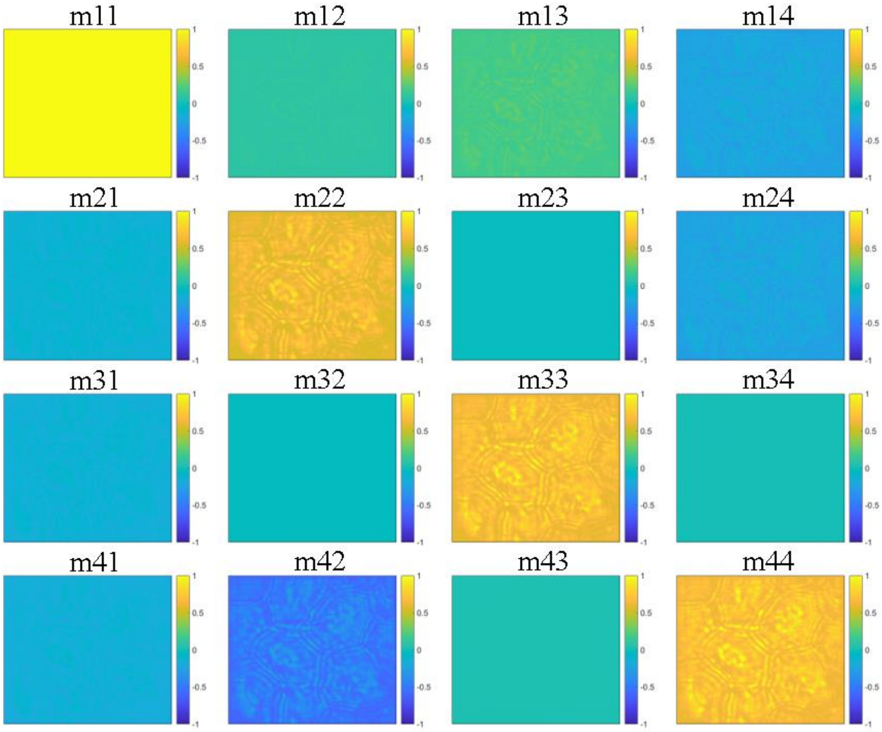


**Fig S5**: Mueller matrix distribution of the same position on the fresh-cut tissue sections of Epipremnum aureum leaf measured at three time points: immediately after sample preparation (0 h), and at 3 h and 6 h intervals.

**F0:**

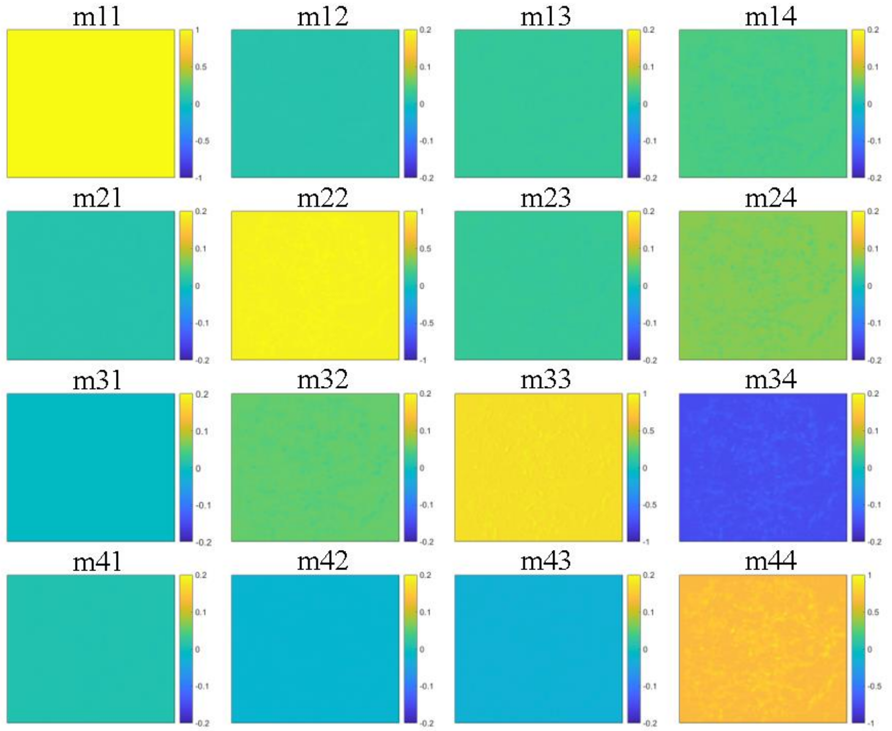
m11
m12
m13
m14
m21
m22
m23
m24
m31
m32
m33
m34
m41
m42
m43
m44

**F2:**

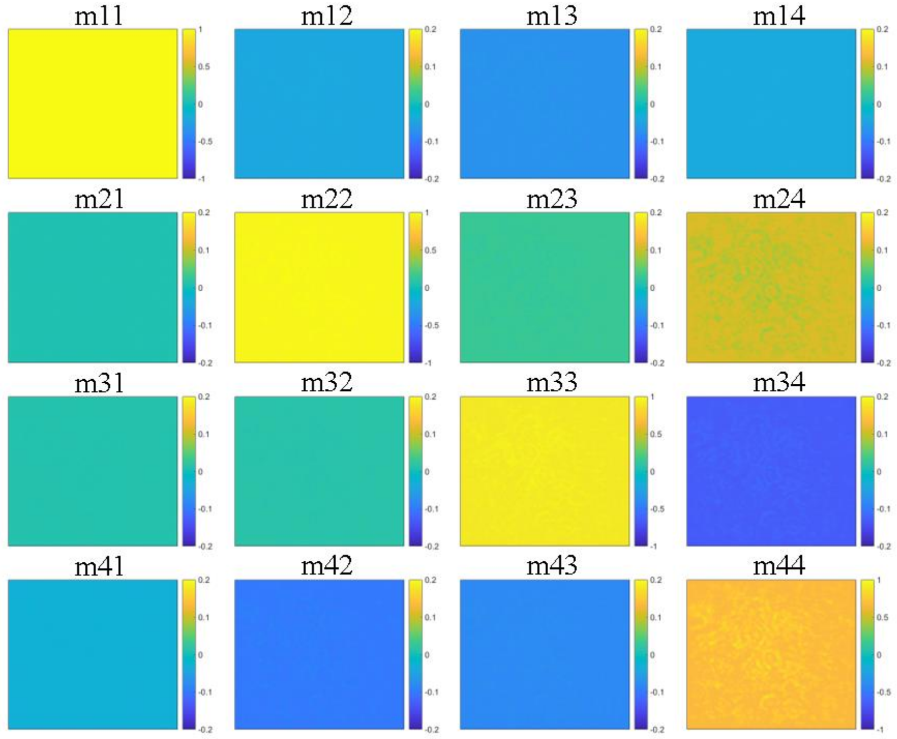
m11
m12
m13
m14
m21
m22
m23
m24
m31
m32
m33
m34
m41
m42
m43
m44

F4:

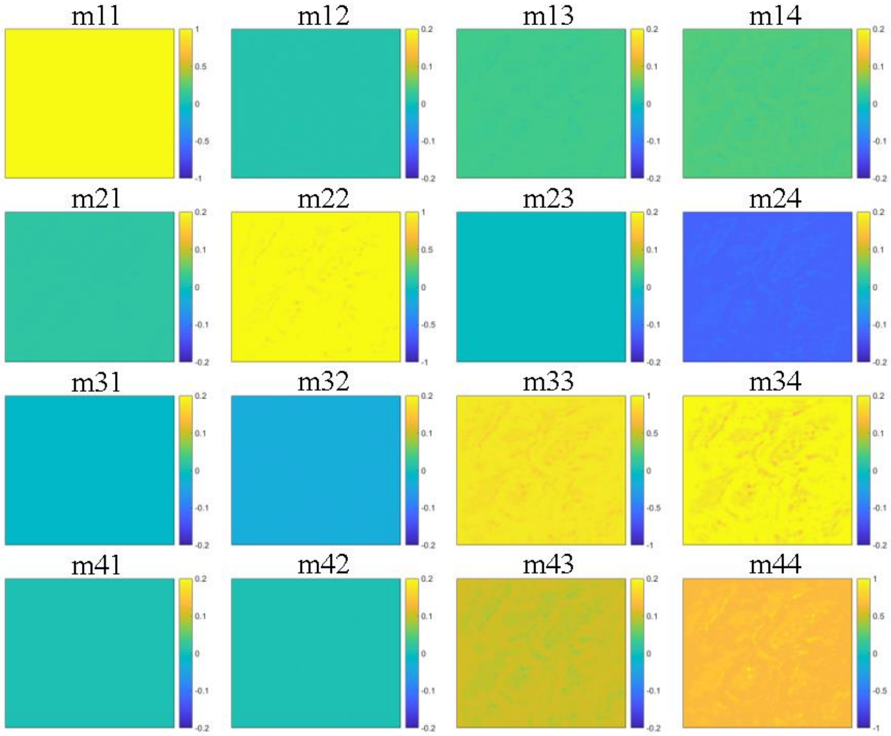


**Fig S6:** The Mueller matrix distribution for each stage of the unstained mouse liver fibrosis tissue paraffin sections: F0（Normal）, F2 (Moderate fibrosis) and F4(Cirrhosis).
